# Using Network Pharmacology and Animal Experiment to Investigate the Therapeutic Mechanisms of Polydatin against Vincristine-Induced Neuropathic Pain

**DOI:** 10.1155/2022/6010952

**Published:** 2022-10-14

**Authors:** Peng Xi, Rui Mao, Shiyan Wu, Lei Liu, Ceng Cai, Lei Lu, Cailin Zhang, Yimei Li

**Affiliations:** ^1^Department of Pain, The First Affiliated Hospital of Xinjiang Medical University, Xinjiang 830054, Urumchi, China; ^2^Department of Tumor Center, The First Affiliated Hospital of Xinjiang Medical University, Xinjiang 830054, Urumchi, China

## Abstract

**Background:**

Polydatin (PD) is the primary active compound in *Polygonum cuspidatum* Sieb and has been demonstrated to exert anti-inflammatory and neuroprotective activities. In the present study, we aimed to explore the therapeutic mechanisms of PD against chemotherapy-induced neuropathic pain.

**Methods:**

The putative targets of PD were obtained from the CTD and SwissTargetPrediction databases. Neuropathic pain- and VIN-related targets were collected from the CTD and GeneCards databases. Subsequently, the intersection targets were obtained using the Venn tool, and the protein-protein interaction (PPI) was constructed by the STRING database. GO and KEGG enrichment analyses were performed to investigate the biological functions of the intersection targets. Further, a rat model of VIN-induced neuropathic pain was established to confirm the reliability of the network pharmacology findings.

**Results:**

A total of 46 intersection targets were identified as potential therapeutic targets, mainly related to neuroinflammation. KEGG pathway analysis indicated that the IL-17 signaling pathway was involved in the mechanism of the antinociceptive effect of PD. PPI network analysis indicated that RELA, IL-6, TP53, MAPK3, and MAPK1 were located at crucial nodes in the network. Additionally, PD exerted an antinociceptive effect by increasing the nociceptive threshold. The results of qRT-PCR, western blot, and immunohisochemistry indicated that PD inhibited the IL-6, TP53, and MAPK1 levels in VIN-induced neuropathic pain rats.

**Conclusions:**

Overall, this research provided evidence that suppressing inflammatory signaling pathways might be a potential mechanism action of PD's antinociceptive effect against VIN-induced neuropathic pain.

## 1. Introduction

Neuropathic pain is a chronic secondary pain, which is characterized by shooting pain or spontaneous persistent and induced magnified pain responses following harmful or non-harmful stimuli [1]. Vincristine (VIN) is a common antineoplastic drug, often used in the treatment of acute lymphoblastic leukemia and Hodgkin's lymphoma. However, a low dose of VIN could cause pain hypersensitivities such as hyperalgesia and allodynia [2]. VIN-induced neuropathic pain is one of the most painful side effects and decreases the life quality of patients [3]. It has been reported that the mechanical actions of neuropathic pain after VIN administration were complex [4]. Recent studies have shown that multiple mechanisms were implicated in the pathologic process of chemotherapy-induced neuropathic pain [5, 6]. Among the pathological factors causing neuropathic pain, neuroinflammation was considered to be one of the major driving factors causing chemotherapy-induced neuropathic pain [5, 7]. In addition, VIN has been revealed to initiate the damage of the blood-nerve barrier and lead to an inflammatory response involving activation of the NF-кB signaling pathway [8]. These proinflammatory cytokines also induced nerve and pain sensitivity and play a key role in maintaining persistent inflammatory pain [9]. Therefore, the efficient control of inflammation is a potential therapy for preventing and treating neuropathic pain.

Polydatin (PD), also known as polygonin, is a stilbenoid originally extracted from the root of *Polygonum cuspidatum* Sieb, a traditional Chinese herbal in China. Previous reports have demonstrated multiple pharmacological effects of PD such as anti-inflammatory, neuroprotective, antioxidative, immune-regulating, cardioprotective, and antiplatelet aggregation activities [10, 11]. PD has been revealed to inhibit dopaminergic neurodegeneration via the inactivation of the NF-кB signaling pathway [12]. PD exhibited its anti-inflammatory effects in BV2 microglia via disrupting lipid rafts [13]. PD also exerted neuroprotective effects in spinal cord injury rats possibly via inhibiting NLRP3 inflammasome activation in the microglia [14]. Moreover, PD was also indicated to promote sciatic nerve repair in diabetic rats via inhibition of RAGE and Keap1 and activation of GLO1 and Nfr2 [15]. PD exerted the anxiolytic effects via suppressing proinflammatory cytokines in a chronic pain mouse model [16]. All these reports suggested that PD may exert therapeutic effects on neuropathic pain. However, there were no studies that investigated the therapeutic effect of PD against chemotherapy-induced neuropathic pain.

Based on these studies, we performed network pharmacology analysis to screen the hub genes and potential therapeutic mechanisms of PD against VIN-induced neuropathic pain. Besides, the analgesic effect of PD associated with the suppression of inflammatory genes level was verified by animal experiments.

## 2. Materials and Methods

### 2.1. Collection of PD-Related Targets

The 2D chemical structure of PD was downloaded from the PubChem Database (https://pubchem.ncbi.nlm.nih.gov/). The comparative toxicogenomics (CTD) database is a premier public resource that advances understanding about human health and chemical exposures [17]. SwissTargetPrediction is a web server for accurately prediction of the potential targets of bioactive compounds [18]. We used CTD (https://ctdbase.org/) and SwissTargetPrediction databases (http://www.swisstargetprediction.ch/) to obtain the PD-related targets.

### 2.2. Collection of Candidate Targets of VIN and Neuropathic Pain

The VIN-related and neuropathic pain-related targets were collected from the CTD database and GeneCards database (https://www.genecards.org/). Then, the intersection targets of PD, VIN, and neuropathic pain were visualized by using the Venn online tool (https://bioinfogp.cnb.csic.es/tools/venny/index.html).

### 2.3. Protein-Protein Interaction (PPI) Network and Network Construction

The intersection genes were uploaded to the String 11.5 (https://cn.string-db.org/), with species limited to “*Homo sapiens*” and the highest confidence (0.9). Then, the TSV format file was downloaded and imported into Cytoscape software (3.8.0) to visualize the PPI network. The key genes were screened according to stress, betweenness, closeness, degree, DMNC, EPC, MNC, and radiality, and the intersection genes were identified as the hub genes. Besides, a compound-targets-pathways network was constructed via the Cytoscape software (3.8.0) [19].

### 2.4. Functional Enrichment Analysis

Metascape could provide a comprehensive gene list annotation and analysis resource for experimental biologists [20]. The intersection targets were imported into the Metascape (https://metascape.org/gp/index.html) to obtain KEGG data and GO biological process data. Then, the clusterProfiler package of R software was used to visualize the results of the KEGG pathway analysis and GO enrichment analysis.

### 2.5. Animal Experimental Protocol

Male Sprague-Dawley rats (190-230 g) were obtained from the Animal Laboratory Center of Xinjiang province and housed under a specific pathogen-free environment (50-60% humidity, 18-22°C temperature, and a 12 h dark/light cycle) with food and water *ad libitum*. The animal experimental protocol was approved by the Animal Experimental Ethics Committee of the First Affiliated Hospital of Xinjiang Medical University, and the experimental procedure was performed by the WHO guidelines for animal care.

Animals were acclimated for one week before induction of chemotherapy pain. VIN was dissolved in sterile saline (0.9% NaCl). The rats from the model group were injected intraperitoneally with VIN (0.1 mg/kg) for ten days (in a two-five days cycle with two days pause) [21], while rats from the control group were injected intraperitoneally with an equal volume of sterile saline (0.9% NaCl). After that, rats from the model group were randomly divided into three groups: the VIN group, rats without drug intervention; low-dose group (VIN+LPD), rats received low dose of PD (15 mg/kg); and high-dose group (VIN + HPD), rats received a high dose of PD (30 mg/kg). PD was administered intraperitoneally daily for 21 days, and the dose was based on a previous study [22]. PD and VIN (purity > 95%) were purchased from Sigma-Aldrich (St. Louis, USA).

### 2.6. Nociceptive Behavioral Tests

Mechanical allodynia was performed on days 0, 7, 14, and 21 according to a previous report [23]. Rats were placed individually in clear plexiglass boxes with a wire mesh floor. Automatic thin steel von Frey filaments were placed below the surface of the rear paw. We gradually increased the force until retracement of the claw was observed and measured the maximum force of the response induced by the mechanical stimulus.

Thermal hyperalgesia was performed on days 0, 7, 14, and 21 according to a previous report [24]. Rats were placed individually in clear plexiglass boxes with a wire mesh floor. A radiative heat source was placed under the surface of the hind paw, and the paw latency reaction times were defined as thermal hyperalgesia. The cut-off point for avoiding tissue injury was set at 20 seconds. The experiment was repeated 3 times in each rat, and the mean value was measured.

### 2.7. Evaluation of Inflammatory Cytokines and Macrophage Marker

After the last nociceptive behavioral tests, the animals were anesthetized with 50 mg/kg of pentobarbital sodium. L4-L6 dorsal root ganglion sections were harvested and homogenized on ice using a homogenizer. Afterward, the homogenate was centrifuged (10,000 g for 10 min, 4°C), and the supernatant was collected. The levels of inflammatory factors (TNF-*α*, IL-6, IL-1*β*, and IL-17) and macrophage marker (CD163) in the supernatant were measured by ELISA kits (Invitrogen, USA) based on the manufacturer's protocols. The catalog numbers of inflammatory cytokines and macrophage marker were as follows: IL-6 (BMS231-2), TNF-*α* (BMS2034), IL-17 (BMS6001TEN), CD163 (88-50361-22), and IL-1*β* (BMS224-2).

### 2.8. Reverse Transcription Quantitative Polymerase Chain Reaction (qRT-PCR)

First, total RNA from the dorsal root ganglion was prepared using the TRIzol reagent (Invitrogen, USA) based on the manufacturer's protocols. Two microgram of RNA was used to synthesize cDNA by cDNA synthesis kits (Invitrogen, USA). qRT-PCR was carried out by using a CFX384 Real-Time System C100 Thermal Cycler (Bio-Rad) based on the manufacturer's protocols. Primer sequences used in the present experiment were presented in Table 1. Relative expression was normalized to the GAPDH using the 2^-*ΔΔ*CT^ method.

### 2.9. Western Blot Analysis

We carried out the western blot analysis based on the standard procedure in previous reports. The dorsal root ganglion tissues were homogenized in cold RIPA lysis buffer with protease inhibitors. After centrifugation at 10,000 g for 20 min, the supernatant was collected for protein quantification. 10 *μ*g of protein was separated by 10% SDS-PAGE and transferred onto the PVDF membrane (Roche). Then, the blot was blocked with defatting milk powder (5%) at room temperature for one hour. After that, the blot was incubated overnight with the following primary antibodies: anti-IL-6 (1 : 1000, Proteintech, USA), anti-MAPK1 (1 : 200, Proteintech, USA), anti-TP53 (1 : 200, Proteintech, USA), and anti-GAPDH (1 : 1000, Proteintech, USA). After incubation, the blots were incubated with horseradish peroxidase-labeled secondary antibody for 60 min at room temperature. Following incubation, the blots were measured by a chemiluminescence reagent (PerkinElmer, USA), and the band intensity quantification was performed using ImageJ software (NIH). GAPDH was used as an endogenous control.

### 2.10. Immunohisochemistry

The dorsal root ganglion tissues were soaked in 4% formalin overnight. 5 *μ*m paraffin sections of dorsal root ganglion was deparaffinized using xylene and rehydrated using a gradient of ethanol. Endogenous peroxidase was suppressed using H_2_O_2_ (3%) for 0.5 h. Then, the slices were incubated with normal goat serum (10%) and anti-IL-6 (1 : 200, Cell Signaling, USA) or anti-TP53 (1 : 200, Cell Signaling, USA) or anti-MAPK1 (1 : 200, Cell Signaling, USA) primary antibodies at 4°C overnight. The slices were washed twice in PBS and incubated with a goat anti-rabbit antibody (1 : 200) at room temperature for 60 min. Subsequently, slices were visualized using a DAB reagent. Finally, we used light microscopy to obtain immunohistochemistry images.

### 2.11. Statistical Analyses

Data were statistically analyzed using GraphPad Prism 5 software and presented as mean ± SD. The results from behavioral tests were analyzed with repeated measures analysis of variance (ANOVA). Comparisons of results between groups were performed using one-way ANOVA followed by Bonferroni's test. Significance was set at *P* < 0.05.

## 3. Results

### 3.1. Collection of PD-Related Targets against VIN-Induced Neuropathic Pain

**Figure 1:**
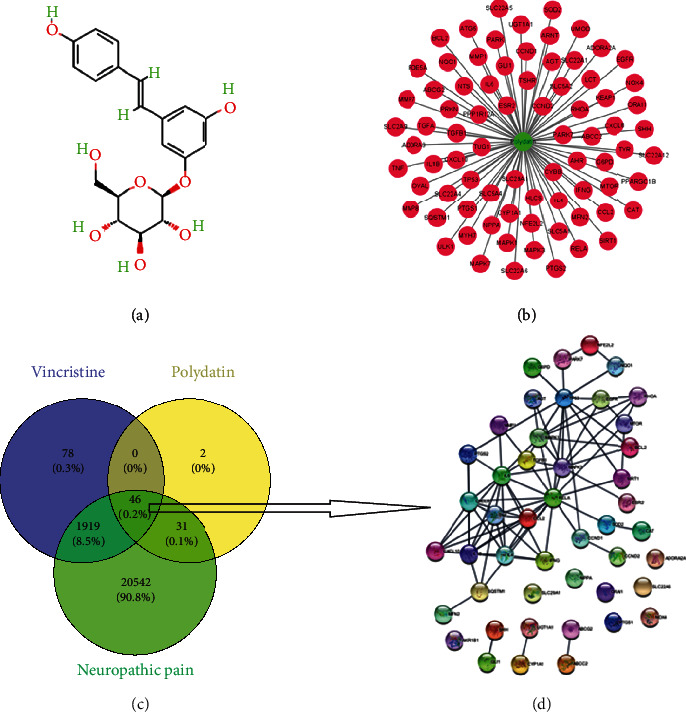
The potential targets of polydatin in the treatment of vincristine-induced neurotoxicity for screening pharmacology biotargets. (a) 2D chemical structure of polydatin. (b) The candidate targets of polydatin. (c) Venn diagram of 46 intersection targets. (d) PPI network of intersection genes generated by STRING.

The PD's 2D structure was shown in Figure 1(a). We used the CTD and SwissTargetPrediction databases and collected 79 PD-related targets (Figure 1(b)). A total of 2043 VIN-related targets and 22538 neurotoxicity-related targets were collected from the CTD and GeneCards databases. Finally, 46 intersection targets were identified as potential therapeutic genes via using a Venn tool (Figure 1(c) and supplementary file 1). The PPI network of intersection genes of PD acting on VIN-induced neurotoxicity was generated by the STRING database (Figure 1(d)).

### 3.2. Functional Analyses of the Potential Targets

**Figure 2:**
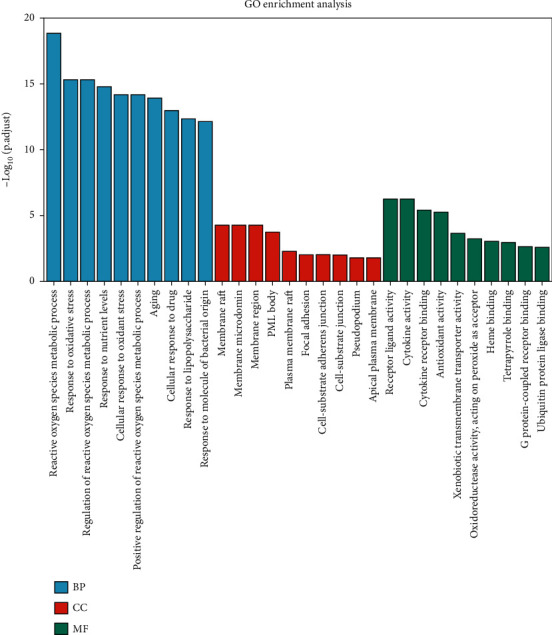
GO enrichment analysis of 46 intersection targets. The top 10 GO terms of biological process (BP), cellular component (CC), and molecular function (MF).

The top 10 GO terms of biological process (BP), cellular component (CC), and molecular function (MF) were presented in Figure 2 and Supplementary file 2, our results revealed that these potential targets were significantly related to reactive oxygen species metabolic process, response to oxidative stress, response to lipopolysaccharide, focal adhesion, cytokine activity, and cytokine receptor binding, etc.

**Figure 3:**
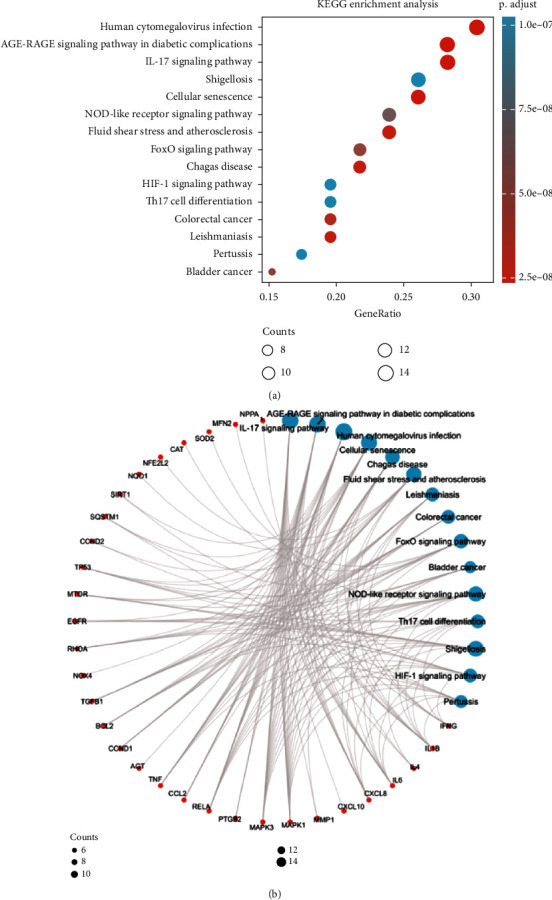
KEGG enrichment analysis of 46 intersection targets. The results of KEGG were presented by bubble (a) and circle (b) charts.

The top 15 most significantly enriched KEGG pathways were presented in Figure 3 and Supplementary file 3, our findings also showed that these potential targets were significantly related to the IL-17 signaling pathway, AGE-RAGE signaling pathway in diabetic complications, cellular senescence, FoxO signaling pathway, NOD-like receptor signaling pathway, Th17 cell differentiation, and HIF-1 signaling pathway, etc.

### 3.3. Identification of Hub Genes

**Figure 4:**
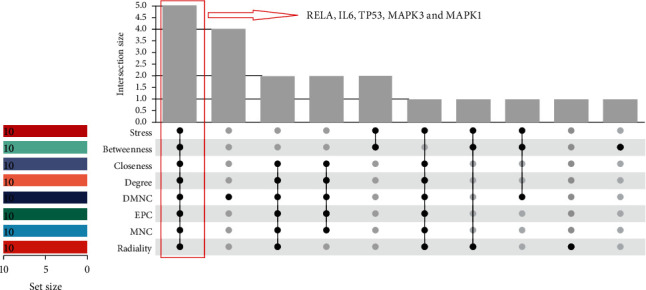
Identification of hub genes. Eight algorithms (stress, betweenness, closeness, degree, DMNC, EPC, MNC, and radiality) were used to identify hub genes based on R package “UpSet.”

The top 10 key genes were selected based on the 8 classification methods (stress, betweenness, closeness, degree, DMNC, EPC, MNC, and radiality) in cytoHubba (Table 2) to screen hub genes. Then, five intersection genes were further screened as hub genes (Figure 4), including RELA, IL-6, TP53, MAPK3, and MAPK1.

### 3.4. Compound-Targets-Pathways Network

**Figure 5:**
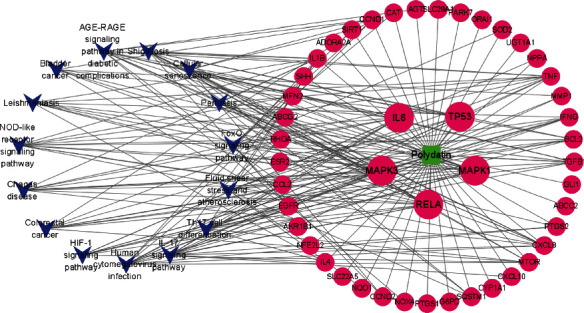
Targets-pathways network of polydatin. Green node represents compound, red nodes represent 46 intersection targets, and blue nodes represent top 15 KEGG signaling pathways.

We constructed a compound-targets-pathways network using Cytoscape software (3.8.0) to obtain a visual analysis result. As shown in Figure 5, the network contains 62 nodes and 203 edges. A green node represents PD, red nodes represent 46 intersection targets, and blue nodes represent the top 15 KEGG signaling pathways. Besides, it was also preliminarily speculated that PD could be used for the treatment of VIN-induced neuropathic pain via the IL-17 signaling pathway, AGE-RAGE signaling pathway in diabetic complications, cellular senescence, FoxO signaling pathway, NOD-like receptor signaling pathway, Th17 cell differentiation, and HIF-1 signaling pathway due to the high representation of RELA, IL-6, TP53, MAPK3, and MAPK1 targets.

### 3.5. PD Inhibited Mechanical Allodynia and Thermal Hyperalgesia Induced by VIN

**Figure 6:**
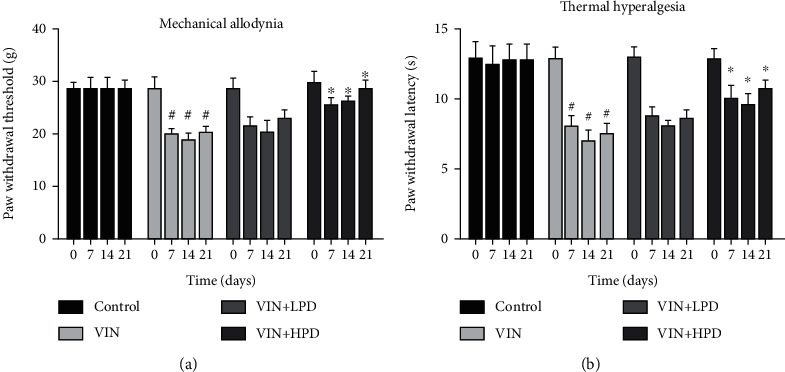
Polydatin relieves pain hypersensitivity in VIN-induced rats. The mechanical allodynia (a) and thermal hyperalgesia (b) were measured in different groups after 0, 7, 14, and 21 days of VIN administration. Results were expressed as mean ± SD (*n* = 6). ^#^*P* < 0.05, the VIN group compared with the control group; ^∗^*P* < 0.05, the VIN+HPD group compared with the VIN-treated group.

As shown in Figure 6, VIN injection-induced mechanical allodynia (20.06 ± 4.61 g) and thermal hyperalgesia (7.51 ± 1.30 s) in the VIN group compared to the control group (*P* < 0.05). A high dose of PD treatment effectively inhibited VIN-induced neuropathic pain via increasing paw withdrawal threshold (28.03 ± 1.87 g) and paw withdrawal latency (10.8 ± 1.43 s) (*P* < 0.05).

### 3.6. PD Alleviated VIN-Induced Inflammatory Response in Dorsal Root Ganglion

**Figure 7:**
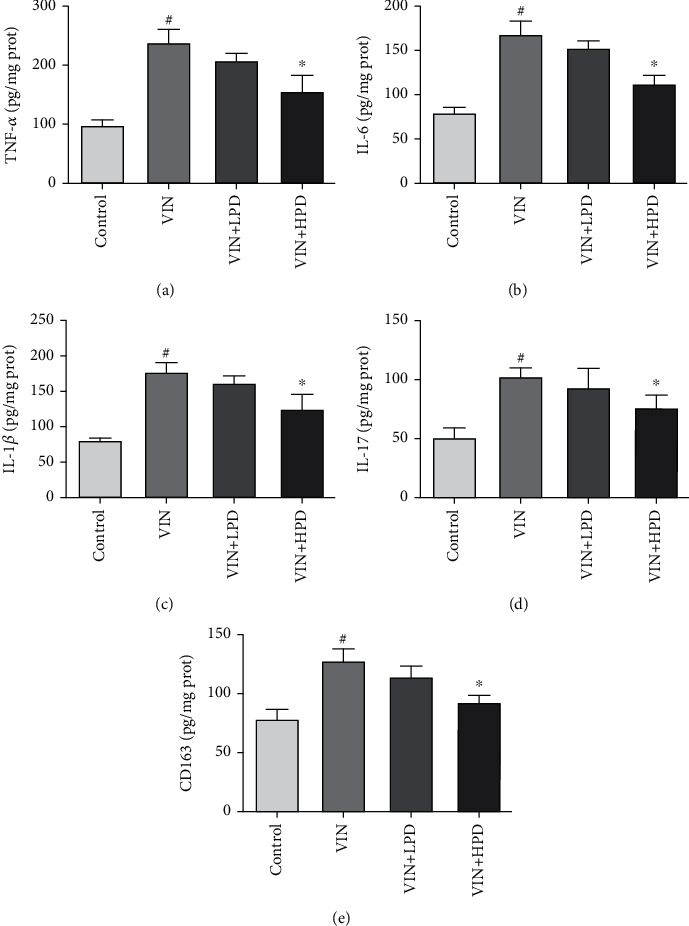
Effect of polydatin on inflammatory cytokines and macrophage marker in VIN-induced rats. The levels of TNF-*α* (a), IL-6 (b), IL-1*β* (c), IL-17 (d), and CD163 (e) in the dorsal root ganglion were measured by ELISA kits. Results were expressed as mean ± SD (*n* = 6). ^#^*P* < 0.05, the VIN group compared with the control group; ^∗^*P* < 0.05, the VIN+HPD group compared with the VIN-treated group.

As shown in Figure 7, VIN injection dramatically increased the levels of TNF-*α* (235.6 ± 23.08 pg/mg prot), IL-6 (166.9 ± 15.36 pg/mg prot), IL-1*β* (175.3 ± 15.41 pg/mg prot), IL-17 (101.1 ± 9.32 pg/mg prot), and CD163 (125.3 ± 11.25 pg/mg prot) in the VIN group compared to those in the control group (*P* < 0.05). A high dose of PD treatment significantly inhibited TNF-*α* (155.9 ± 27.87 pg/mg prot), IL-6 (111.4 ± 11.75 pg/mg prot), IL-1*β* (125.2 ± 21.13 pg/mg prot), IL-17 (74.98 ± 12.51 pg/mg prot), and CD163 (92.10 ± 7.92 pg/mg prot) in VIN+HPD compared to those in the VIN group (*P* < 0.05).

### 3.7. Effect of PD on Hub Expression in VIN-Induced Neuropathic Pain Rats

**Figure 8:**
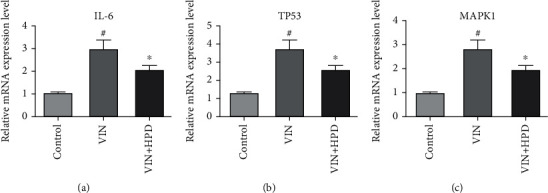
Validation of hub genes. qRT-PCR analysis for differences in mRNA expressions of IL-6 (a), TP53 (b), and MAPK1 (c) in the different groups. Results were expressed as mean ± SD (*n* = 3). ^#^*P* < 0.05, the VIN group compared with the control group; ^∗^*P* < 0.05, the VIN+HPD group compared with the VIN-treated group.

**Figure 9:**
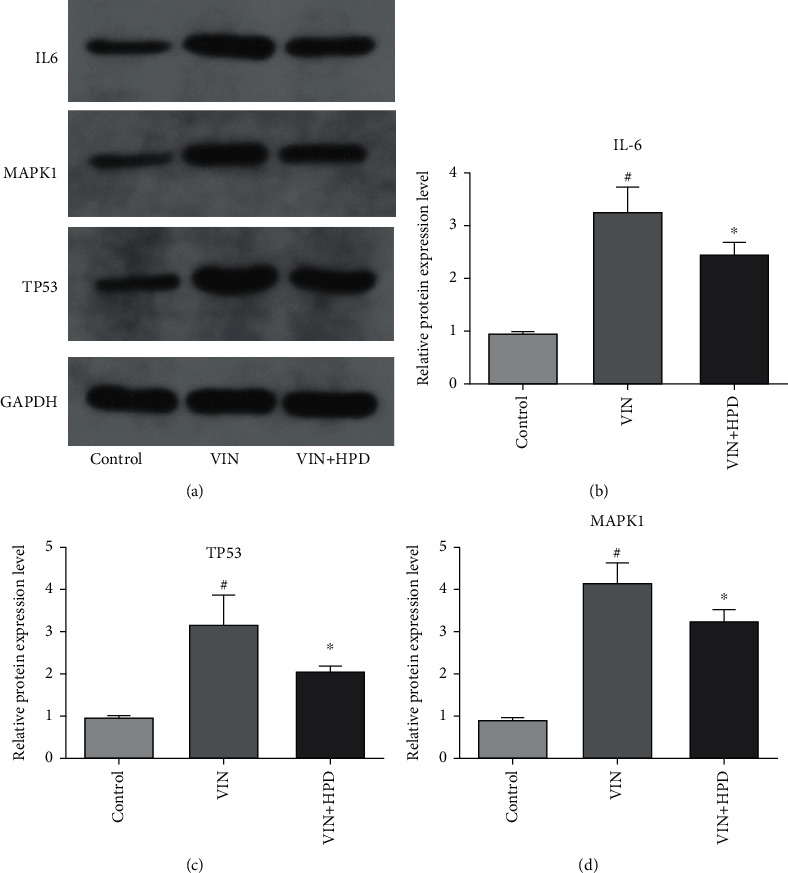
Validation of hub genes via western blot analyses. (a) Representative western blot analyses of IL-6, MAPK1, TP53, and GAPDH. The normalized optical density of IL-6 (b), TP53 (b), and MAPK1 (d). Results were expressed as mean ± SD (*n* = 3). ^#^*P* < 0.05, the VIN group compared with the control group; ^∗^*P* < 0.05, the VIN+HPD group compared with the VIN-treated group.

**Figure 10:**
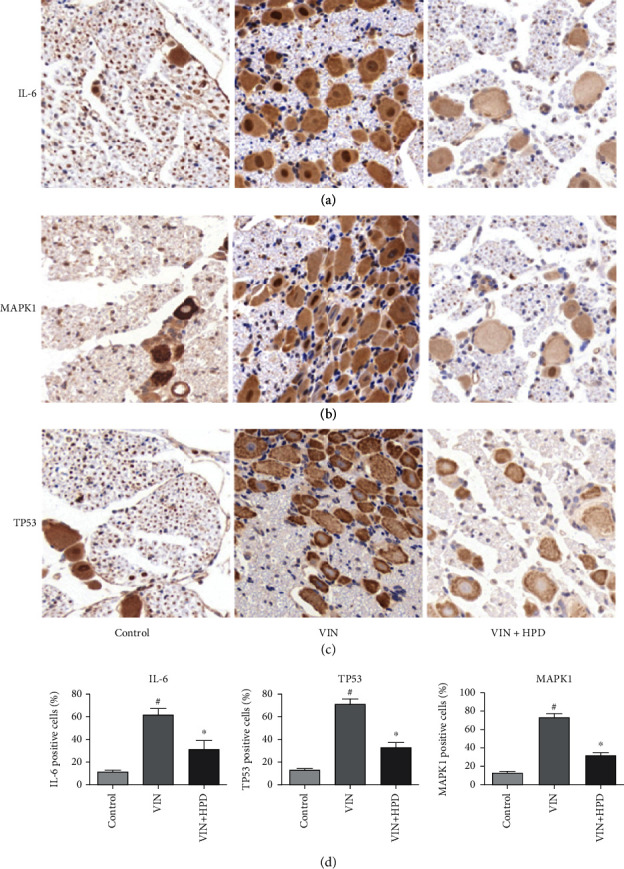
Immunohistochemical staining of IL-6 (a), MAPK1 (b), and TP53 (c) in the dorsal root ganglion. (d) The percentage of IL-6-, TP53-, and MAPK1-positive cells in different groups. ^#^*P* < 0.05, the VIN group compared with the control group; ^∗^*P* < 0.05, the VIN+HPD group compared with the VIN-treated group.

We verified the expression of the three hub genes using qRT-PCR analysis and western blot analysis to further clarify the potential mechanism of PD against VIN-induced neurotoxicity. As shown in Figure 8, VIN injection upregulated the mRNA expression of IL-6 (2.9 ± 0.37), TP53 (3.36 ± 0.58), and MAPK1 (2.76 ± 0.41) in the VIN group compared to those in the control group (*P* < 0.05). A high dose of PD treatment downregulated the expression of IL-6 (2.03 ± 0.21), TP53 (2.08 ± 0.54), and MAPK1 (1.66 ± 0.34) in the VIN+HPD group (*P* < 0.05). Besides, VIN injection upregulated the protein expression of IL-6 (3.23 ± 0.51), TP53 (3.16 ± 0.70), and MAPK1 (4.16 ± 0.45) in the VIN group compared to those in the control group (*P* < 0.05) (Figure 9). A high dose of PD reversed those changes induced by VIN (*P* < 0.05), which was consistent with the above findings. We also performed the immunohistochemical experiment to further validate the above results. As shown in Figure 10, the expression levels of IL-6 (61.33 ± 5.71%), TP53 (71.70 ± 4.34%), and MAPK1 (72.73 ± 4.18%) in the VIN group were obviously upregulated (*P* < 0.05). However, the expression levels of IL-6 (31.30 ± 7.59%), TP53 (32.97 ± 5.35%), and MAPK1 (31.73 ± 2.95%) in the VIN+HPD group were lower than those of the VIN group (*P* < 0.05).

## 4. Discussion

Chemotherapy-induced neuropathic pain is a complex chronic disease, which is caused by damage to the nervous system [25]. Chemotherapy-induced neuropathic pain also can lead to loss of functional capacity and negatively affect the quality of life, resulting in lower doses of chemotherapy drugs, and ultimately, impacting the overall survival rates of patients [26]. Therefore, it is necessary to explore novel therapeutic strategies for the treatment of chemotherapy-induced neuropathic pain. Recently, some studies have demonstrated that natural compounds exerted analgesic effects with little adverse effects, suggesting that plant-derived natural products have a therapeutic potential for developing new drugs in the treatment of neuropathic pain [27, 28]. PD is a stilbenoid and has been reported to inhibit apoptosis, inflammation, and oxidative stress as the main pathway for neurodegenerative diseases [11]. PD has been revealed to exert neuroprotective effects in spinal cord injury rats via inhibiting microglial inflammation [14]. Furthermore, PD had the anxiolytic effects via inhibiting inflammatory cytokines in a chronic pain mouse model [16]. These studies implied that PD may have pharmacological effects against pain. However, the therapeutic effect of PD against neuropathic pain remains unclear. In recent years, some researchers used network pharmacology analysis to screen and confirm the active ingredients and potential therapeutic targets. This method provides a powerful tool for elucidating the mechanisms of disease and facilitating the discovery of potential active ingredients [29].

In the present study, the network pharmacology and animal experimental approach were performed to reveal the potential therapeutic mechanisms of PD in the treatment of VIN-induced neurotoxicity. First, we identified 46 potential targets using the public databases. The results of KEGG enrichment analysis showed that these target genes were mainly involved in the IL-17 signaling pathway and cytokine-cytokine receptor interaction, which were closely associated with the pathologic process of chemotherapy-induced neuropathic pain. Based on the results of GO-BP enrichment analysis, these target genes mainly focused on the cellular response to lipopolysaccharide, cytokine-mediated signaling pathway, positive regulation of leukocyte cell-cell adhesion, humoral immune response, neuroinflammatory response, etc. In addition, we also identified five hub genes (RELA, IL-6, TP53, MAPK3, and MAPK1) as the most promising candidate targets of PD acting on the progression of chemotherapy-induced neuropathic pain.

Neuroinflammation is a potential codriver of chemotherapy-induced neuropathic pain. It has been reported that the chemotherapy-induced increase in inflammatory factors and a close relationship with the occurrence and development of neuropathic pain [8, 30]. Macrophage infiltration causes a subsequent secretion and generation of various chemokines (CXC family) and inflammatory cytokines (IL-6, IL-1*β*, and TNF-*α*) [31]. These molecules were thought to be potential drivers for the initiation and development of neuropathic pain [32]. In the peripheral nervous system, proinflammatory cytokines not only modulate the sensitivity and activity of spontaneous nociceptors but also promote axonal injury via activation of inflammation [33]. Besides, a previous report revealed that IL-8 and IL-6 mRNA expressions were upregulated in suffering from neuropathic pain [34]. IL-17 is an important mediator of inflammatory responses and was implicated in evoking proinflammatory reactions. It has been demonstrated that IL-17 could promote neuroinflammation and pain hypersensitivity following peripheral nerve damage [35]. Moreover, IL-17 could mediate neuronal hyperexcitability and neuron-glial interactions in chemotherapy-induced neuropathic pain [36]. To support these findings, spironolactone, an aldosterone receptor antagonist with anti-inflammatory effects has been shown to have beneficial effects in ameliorating VIN-induced neuropathic pain [37]. In our study, KEGG enrichment analysis indicated that most of the targets were enriched in the inflammation-related signaling pathway, such as the IL-17 signaling pathway. Besides, IL-6 was identified as the hub gene in the PPI network. Our *in vivo* experiment further indicated that PD decreased the gene expression of IL-6 in VIN-induced neuropathic pain, implying that IL-6 was the potential therapeutic target of PD against VIN-induced neuropathic pain.

TP53, a major neuronal proapoptotic gene, was implicated in synaptic terminal injury and apoptosis, and its activation was associated with the etiopathogenesis of Parkinson's disease [38, 39]. It has been demonstrated that the TP53 gene regulated dopaminergic neuronal injury in different neurotoxicant models [40]. In addition, suppression of the TP53 gene via using a dominant-negative form of TP53 or pharmacological inhibitors exerted protection to endogenous dopamine neurons [41]. Moreover, previous reports have revealed that TP53 and MAP2K2 might be involved in the pathological process of neuropathic pain via bioinformatics analysis [42, 43]. MAPK1, an extracellular signal-regulated kinase, was involved in various cellular processes. MAPK activation plays an important role in the pathophysiology of spinal cord injury, and inhibition of the MAPK3/MAPK1 signaling pathway might be effective in the treatment of inflammation, trauma, and spinal cord injury [44]. The previous report has indicated that the knockdown of NEAT1 inhibited the inflammation of spinal cord injury via the miR-211-5p/MAPK1 axis [45]. In the present study, TP53 and MAPK1 were identified as hub genes in the PPI network. Our *in vivo* experiment further indicated that PD regulated the gene expressions of TP53 and MAPK1 in VIN-induced neuropathic pain, suggesting that TP53 and MAPK1 were the potential therapeutic targets of PD against VIN-induced neurotoxicity. One of the limitations of this study is the species difference between rodent pain models and clinical chemotherapy-induced neuropathic pain. To overcome this hurdle, the use of species that are closer to humans than rodents, such as nonhuman primates, could hence understand the neuropathic pain and improve the passage of new therapies through clinical testing.

## 5. Conclusion

In conclusion, our study firstly demonstrated the therapeutic effects of PD on VIN-induced neurotoxicity via network pharmacology and experimental verification. These findings indicated that PD alleviated VIN-induced neurotoxicity via downregulation of IL-6, TP53, and MAPK1 expressions. This study provided a novel approach to exploring the potential therapeutic mechanism of PD in the treatment of neuropathic pain.

## Figures and Tables

**Table 1:** Sequences of primers used quantitative real-time PCR.

Gene	Forward primer (5′ to 3′)	Reverse primer (5′ to 3′)
MAPK1	GGTTGGGTTTGGGTTTGGTTCATTG	CGCTGCCTTCTGCTGCTTCTAC
TP53	GAAGACTCCAGGTAGGAAGC	GTCTCTCCCAGGACAGGTA
IL-6	TCCTACCCCAACTTCCAATGCTC	TTGGATGGTCTTGGTCCTTAGCC
GAPDH	CTGACGAAGGACAATGAGTGCACAGCGC	ATTCCACATCACAAGACTTCGCTCAGCC

**Table 2:** The top 10 gene rank in cytoHubba.

	Rank methods in cytoHubba							
	Radiality	MNC	EPC	DMNC	Degree	Closeness	Betweenness	Stress
Top 10 genes	IL-1*β*	IL-1*β*	IL-1*β*	BCL2	IL-1*β*	IL-1*β*	SQSTM1	SQSTM1
	IL-6	IL-6	IL-6	IL-1*β*	IL-6	IL-6	TP53	IL-1*β*
	TP53	TP53	TP53	SQSTM1	TP53	TP53	IL-6	TP53
	CXCL8	CXCL8	CXCL8	PTGS2	CXCL8	CXCL8	NQO1	IL-6
	CCND1	CCL2	CCL2	CXCL8	CCL2	CCL2	PARK7	NQO1
	RELA	RELA	RELA	CCL2	RELA	RELA	SOD2	PARK7
	TGFB1	IL-4	IL-4	IFNG	IL-4	IL-4	CCND1	CCND1
	MAPK1	MAPK1	MAPK1	IL-4	MAPK1	MAPK1	RELA	RELA
	MAPK3	MAPK3	MAPK3	CXCL10	MAPK3	MAPK3	MAPK1	MAPK1
	TNF	TNF	TNF	TNF	TNF	TNF	MAPK3	MAPK3

## Data Availability

The data used to support the study are available from the corresponding author.

## Supplementary Materials

Supplementary 1 Supplementary file 1: the potential targets of polydatin in the treatment of vincristine-induced neurotoxicity.

Supplementary 2 Supplementary file 2: the top 10 GO terms of biological process (BP), cellular component (CC), and molecular function (MF).

Supplementary 3 Supplementary file 3: the top 15 most significantly enriched KEGG pathways.
