# Supplementary material for: Using Network Pharmacology and Animal Experiment to Investigate the Therapeutic Mechanisms of Polydatin against Vincristine-Induced Neuropathic Pain
Source: Mediators Inflamm. 2022 Oct 14;2022:6010952. doi: 10.1155/2022/6010952 (PMC9587674; doi:10.1155/2022/6010952)
Supplement: Supplementary 2 — Supplementary file 2: the top 10 GO terms of biological process (BP), cellular component (CC), and molecular function (MF). [file 6010952.f2.pdf]

| Ontology | ID | Description | GeneRatio | BgRatio | pvalue | p.adjust | qvalue |
|---|---|---|---|---|---|---|---|
| BP | GO:0072593 | reactive oxygen species metabolic process | 19/46 | 284/18670 | 4.54e-23 | 1.55e-19 | 5.04e-20 |
| BP | GO:0006979 | response to oxidative stress | 19/46 | 451/18670 | 2.97e-19 | 4.89e-16 | 1.59e-16 |
| BP | GO:2000377 | regulation of reactive oxygen species metabolic process | 15/46 | 195/18670 | 4.29e-19 | 4.89e-16 | 1.59e-16 |
| BP | GO:0031667 | response to nutrient levels | 19/46 | 499/18670 | 1.97e-18 | 1.68e-15 | 5.46e-16 |
| BP | GO:0034599 | cellular response to oxidative stress | 16/46 | 302/18670 | 9.46e-18 | 6.47e-15 | 2.10e-15 |
| BP | GO:2000379 | positive regulation of reactive oxygen species metabolic process | 12/46 | 102/18670 | 1.21e-17 | 6.90e-15 | 2.24e-15 |
| BP | GO:0007568 | aging | 16/46 | 321/18670 | 2.50e-17 | 1.22e-14 | 3.96e-15 |
| BP | GO:0035690 | cellular response to drug | 16/46 | 369/18670 | 2.27e-16 | 9.69e-14 | 3.15e-14 |
| BP | GO:0032496 | response to lipopolysaccharide | 15/46 | 330/18670 | 1.17e-15 | 4.43e-13 | 1.44e-13 |
| BP | GO:0002237 | response to molecule of bacterial origin | 15/46 | 343/18670 | 2.07e-15 | 7.06e-13 | 2.29e-13 |
| CC | GO:0045121 | membrane raft | 8/46 | 315/19717 | 5.98e-07 | 5.51e-05 | 4.15e-05 |
| CC | GO:0098857 | membrane microdomain | 8/46 | 316/19717 | 6.12e-07 | 5.51e-05 | 4.15e-05 |
| CC | GO:0098589 | membrane region | 8/46 | 328/19717 | 8.11e-07 | 5.51e-05 | 4.15e-05 |
| CC | GO:0016605 | PML body | 5/46 | 99/19717 | 3.36e-06 | 1.71e-04 | 1.29e-04 |
| CC | GO:0044853 | plasma membrane raft | 4/46 | 109/19717 | 1.21e-04 | 0.005 | 0.004 |
| CC | GO:0005925 | focal adhesion | 6/46 | 405/19717 | 3.38e-04 | 0.009 | 0.007 |
| CC | GO:0005924 | cell-substrate adherens junction | 6/46 | 408/19717 | 3.52e-04 | 0.009 | 0.007 |
| CC | GO:0030055 | cell-substrate junction | 6/46 | 412/19717 | 3.71e-04 | 0.009 | 0.007 |
| CC | GO:0031143 | pseudopodium | 2/46 | 17/19717 | 7.08e-04 | 0.016 | 0.012 |
| CC | GO:0016324 | apical plasma membrane | 5/46 | 318/19717 | 8.43e-04 | 0.016 | 0.012 |

| Ontology | ID | Description | GeneRatio | BgRatio | pvalue | p.adjust | qvalue |
|---|---|---|---|---|---|---|---|
| MF | GO:0048018 | receptor ligand activity | 12/46 | 482/17697 | 2.44e-09 | 5.72e-07 | 3.79e-07 |
| MF | GO:0005125 | cytokine activity | 9/46 | 220/17697 | 4.45e-09 | 5.72e-07 | 3.79e-07 |
| MF | GO:0005126 | cytokine receptor binding | 9/46 | 286/17697 | 4.33e-08 | 3.71e-06 | 2.46e-06 |
| MF | GO:0016209 | antioxidant activity | 6/46 | 86/17697 | 8.85e-08 | 5.68e-06 | 3.77e-06 |
| MF | GO:0042910 | xenobiotic transmembrane transporter activity | 3/46 | 13/17697 | 4.62e-06 | 2.37e-04 | 1.57e-04 |
| MF | GO:0016684 | oxidoreductase activity, acting on peroxide as acceptor | 4/46 | 56/17697 | 1.33e-05 | 5.69e-04 | 3.78e-04 |
| MF | GO:0020037 | heme binding | 5/46 | 135/17697 | 2.56e-05 | 9.39e-04 | 6.23e-04 |
| MF | GO:0046906 | tetrapyrrole binding | 5/46 | 145/17697 | 3.60e-05 | 0.001 | 7.68e-04 |
| MF | GO:0001664 | G protein-coupled receptor binding | 6/46 | 280/17697 | 8.19e-05 | 0.002 | 0.002 |
| MF | GO:0031625 | ubiquitin protein ligase binding | 6/46 | 290/17697 | 9.93e-05 | 0.003 | 0.002 |
