# Supplementary material for: Using Network Pharmacology and Animal Experiment to Investigate the Therapeutic Mechanisms of Polydatin against Vincristine-Induced Neuropathic Pain
Source: Mediators Inflamm. 2022 Oct 14;2022:6010952. doi: 10.1155/2022/6010952 (PMC9587674; doi:10.1155/2022/6010952)
Supplement: Supplementary 3 — Supplementary file 3: the top 15 most significantly enriched KEGG pathways. [file 6010952.f3.pdf]

Ontology	ID	Description	GeneRatio	BgRatio	pvalue	p.adjust	qvalue
KEGG	hsa04657	IL-17 signaling pathway	13/46	94/8076	2.27e-15	4.78e-13	1.29e-13
KEGG	hsa04933	AGE-RAGE signaling pathway in diabetic complications	13/46	100/8076	5.25e-15	5.51e-13	1.49e-13
KEGG	hsa05163	Human cytomegalovirus infection	14/46	225/8076	1.24e-11	8.65e-10	2.34e-10
KEGG	hsa04218	Cellular senescence	12/46	156/8076	3.90e-11	2.05e-09	5.54e-10
KEGG	hsa05142	Chagas disease	10/46	102/8076	1.84e-10	7.36e-09	1.99e-09
KEGG	hsa05418	Fluid shear stress and atherosclerosis	11/46	139/8076	2.10e-10	7.36e-09	1.99e-09
KEGG	hsa05140	Leishmaniasis	9/46	77/8076	3.34e-10	1.00e-08	2.72e-09
KEGG	hsa05210	Colorectal cancer	9/46	86/8076	9.18e-10	2.41e-08	6.52e-09
KEGG	hsa04068	FoxO signaling pathway	10/46	131/8076	2.22e-09	4.93e-08	1.34e-08
KEGG	hsa05219	Bladder cancer	7/46	41/8076	2.35e-09	4.93e-08	1.34e-08
KEGG	hsa04621	NOD-like receptor signaling pathway	11/46	181/8076	3.57e-09	6.81e-08	1.84e-08
KEGG	hsa04659	Th17 cell differentiation	9/46	107/8076	6.56e-09	1.08e-07	2.94e-08
KEGG	hsa05131	Shigellosis	12/46	246/8076	7.58e-09	1.08e-07	2.94e-08
KEGG	hsa04066	HIF-1 signaling pathway	9/46	109/8076	7.74e-09	1.08e-07	2.94e-08
KEGG	hsa05133	Pertussis	8/46	76/8076	8.27e-09	1.08e-07	2.94e-08
